# Effects of Potassium Levels on Plant Growth, Accumulation and Distribution of Carbon, and Nitrate Metabolism in Apple Dwarf Rootstock Seedlings

**DOI:** 10.3389/fpls.2020.00904

**Published:** 2020-06-23

**Authors:** Xinxiang Xu, Xin Du, Fen Wang, Jianchuan Sha, Qian Chen, Ge Tian, Zhanling Zhu, Shunfeng Ge, Yuanmao Jiang

**Affiliations:** State Key Laboratory of Crop Biology, College of Horticulture Science and Engineering, Shandong Agricultural University, Tai’an, China

**Keywords:** M9T337 seedlings, biomass, enzyme activity, NUE, ^15^N, ^13^C, nitrate transporter gene expression

## Abstract

Nitrogen (N) is one of the most required mineral elements for plant growth, and potassium (K) plays a vital role in nitrogen metabolism, both elements being widely applied as fertilizers in agricultural production. However, the exact relationship between K and nitrogen use efficiency (NUE) remains unclear. Apple dwarf rootstock seedlings (M9T337) were used to study the impacts of different K levels on plant growth, nitrogen metabolism, and carbon (C) assimilation in water culture experiments for 2 years. The results showed that both deficiency and excess K inhibited the growth and root development of M9T337 seedlings. When the K supply concentration was 0 mM and 12 mM, the biomass of each organ, root-shoot ratio, root activity and NO_3_^–^ ion flow rate decreased significantly, net photosynthetic rate (*P*_n_) and photochemical efficiency (*F*_v_/*F*_m_) being lower. Meanwhile, seedlings treated with 6 mM K^+^ had higher N and C metabolizing enzyme activities and higher nitrate transporter gene expression levels (*NRT1.1*; *NRT2.1*). ^13^C and ^15^N labeling results showed that deficiency and excess K could not only reduce ^15^N absorption and ^13^C assimilation accumulation of M9T337 seedlings, but also reduced the ^15^N distribution ratio in leaves and ^13^C distribution ratio in roots. These results suggest that appropriate K supply (6 mM) was optimal as it enhanced photoassimilate transport from leaves to roots and increased NUE by influencing photosynthesis, C and N metabolizing enzyme activities, nitrate assimilation gene activities, and nitrate transport.

## Introduction

Nitrogen (N) is one of the most abundant elements on the earth and plays an important role in modern agriculture (Chen et al., 2018). N has an irreplaceable role in organ construction, material metabolism, fruit yield, and the quality formation of fruit trees (Warner et al., 2004; Liu et al., 2010; Bai et al., 2016). Sufficient N application not only improves the photosynthetic efficiency of the leaves but also promotes flower bud differentiation (Li et al., 2012; Wei et al., 2016; Liu et al., 2018), enhances fruit setting rate, and increases yield (Raese et al., 2007). Due to the one-sided pursuit of high yield and large fruits by fruit farmers, the excessive application of N fertilizer in apple orchards has become common in China. The applied N fertilizer dose is are more above the demand of the tree (Ge et al., 2018). Furthermore, the over application of N increases the input cost of farmers, decreases fruit yield and quality (Chen et al., 2017), and directly leads to a decreased N utilization rate. Excessive N also indirectly results in adverse ecological effects, including the increased N deposition, the intensification of the greenhouse effect, soil acidification, and the eutrophication of water bodies (Liu et al., 2013; Zhu and Zhang, 2017). Therefore, it is a main issue for Chinese apple production to promote the efficient utilization of N to improve the quality and yield of apple.

Potassium (K) is the most abundant inorganic cation, and it is important for ensuring optimal plant growth (White and Karley, 2010). K is an activator of dozens of important enzymes, such as protein synthesis, sugar transport, N and C metabolism, and photosynthesis. It plays an important role in the formation of yield and quality improvement (Marschner, 2012; Oosterhuis et al., 2014). K is also very important for cell growth, which is an important process for the function and development of plants (Hepler et al., 2001). In terms of the growth-promoting mechanism of K, it is generally agreed that K stimulates and controls ATPase in the plasma membrane to generate acid stimulation, which then triggers cell wall loosening and hydrolase activation (Oosterhuis et al., 2014), thus promoting cell growth. K has strong mobility in plants and plays an important role in regulating cell osmotic pressure and balancing the cations and anions in the cytoplasm (Kaiser, 1982; Hu et al., 2016a). Through these processes, K is involved in the regulation of stomatal opening and closing, cell elongation, and other important physiological processes. There are many studies on the effect of K level on plant growth. Jin et al. (2007) found that the highest yield and fruit quality were obtained in Red Fuji apple under treatment with 600 kg/ha K; Wang et al. (2017) determined that 6 mM K treatment promoted pear growth and improved photosynthetic efficiency; and Lu et al. (2001) also reported increased production with better fruit quality parameters in navel orange supplied under 500 kg/ha K. There is an interaction between K and other nutrient ions. High K concentrations in the soil solution inhibit Mg uptake and may induce Mg deficiency in plants (Tränkner et al., 2018). However, K deficiency could promote the absorption of Na^+^ and Ca^2+^ in maize (Du et al., 2017), and could inhibit N absorption in cotton and significantly reduce the content of NO_3_^–^ in the leaves (Hu et al., 2017). It is evident that K affects significantly the absorption and utilization of other nutrients by plants, and the appropriate K level differs in different crops.

Among the interactions between K and other nutrients, the interaction with N is the most important. Some studies evaluated the relationship between K and N metabolism. In contrast to the antagonistic relationship between K^+^ and NH_4_^+^ nutrition, the acquisition rates of K^+^ and NO_3_^–^ are often found to be positively correlated (Rufty et al., 1981; Coskun et al., 2016), and sufficient K supply can promote N metabolism and enhance the synthesis of amino acids and proteins (Ruan et al., 1998; Ruiz and Romero, 2002). Hu et al. (2016b) found that K deficiency could reduce Nitrate reductase (NR), Glutamine synthetase (GS), and Glutamate synthase (GOGAT) activities and inhibit nitrate absorption in cotton, whereas Armengaud et al. (2009) found that K deficiency could up-regulate the activities of GS and Glu dehydrogenase (GDH) in *Arabidopsis.* Metabolism of N affected by K appears to vary in different types of plants. Meanwhile, the level of K has a significant impact on C metabolism, and also a strong interaction exists between C metabolism and N metabolism in the metabolic process and energy level (Hu et al., 2017). Based on previous studies, we further evaluated the effects of different K levels on photosynthesis, C metabolism, nitrate uptake, utilization and distribution of M9T337 seedlings through non-invasive micro-measurement technology, ^15^N, ^13^C isotope labeling, and fluorescence quantitative PCR technology. Our studies provided strong evidence for the direct or indirect impact of K level on N absorption and utilization. These experimental results will provide a scientific basis toward the amelioration of problems, related to the poor growth and low N utilization rate of M9T337 seedlings caused by unreasonable K application.

## Materials and Methods

### Plant Materials and Treatments

The experiment was conducted in 2018 and 2019. M9T337 seedlings, an apple dwarf rootstock, were used in current study. M9T337 seedlings (*n* = 200) were grown under natural light, 22–28°C (day) and 5–10°C (night), and a relative humidity (RH) of 55–65%. Seedlings with eight true leaves and about 15 cm height (about 60 days old) were planted on foam plates with eight holes in each plate. Single seedling was planted in each hole. Six liters of nutrient solution was added to each basin (plate). The seedlings were cultured with ^1^/_2_ Hoagland’s (Hoagland and Arnon, 1950) nutrient solution for 7 days to gradually adapt to the nutrient solution, following which they were transferred to full-concentration Hoagland’s nutrient solution. After 30 days of treatment, the NO_3_^–^ ion velocity of the root system was measured, and the root morphology, root activity, biomass of each organ, and K content were measured.

The trial began on April 9 (10 days after planted), with K_2_SO_4_ as the only K source. Five K supply levels (0, 3, 6, 9, and 12 mM) were applied with 16 repetitions, respectively. The specific concentrations of the other nutrients were as follows: 4 mM Ca(NO_3_)_2_, 1 mM Ca(^15^NO_3_)_2_ [Ca(^15^NO_3_)_2_, produced by the Shanghai Institute of Chemical Technology, with abundances of 10.14%, for ^15^N marking], 2 mM MgSO_4_, 1 mM NaH_2_PO_4_, 1 mM EDTA-Fe, 37 μM H_3_BO_4_, 9 μM MnCl_2_ ⋅ 4H_2_O, 0.3 μM CuSO_4_ ⋅ 5H_2_O, and 0.76 μM ZnSO_4_ ⋅ 7H_2_O. The pH of the nutrient solution was adjusted to 6.0 ± 0.1 with H_3_PO_4_ or NaOH. The test processing time was 30 days. Samples were taken at 9:00–10:00 a.m. on the 10th, 20th, and 30th day of treatment for enzyme activity and nitrate transporter gene determination. After 30 days of treatment, the NO_3_^–^ ion velocity of the root system was measured, and the root morphology, root activity, biomass of each organ, K content, and ^15^N abundance were measured.

### ^13^C Labeling of Seedlings and Isotope Analysis

After 27 days of treatment, ^13^C isotope labeling was performed. Ba^13^CO_3_ (^13^C independence is 98%) was used as a selection marker, and the dosage was 0.2 g. The seedlings were placed together with the markers, fans, and reduced iron powder into a sealed marking room to make transparent film. The transmittance of sunlight in the labeling chamber was 95% of the natural light intensity. Labeling work started at 9:00 a.m., at which point the fan was turned on and the labeling chamber was sealed. One milliliter of hydrochloric acid (1 mol/L) was injected into the beaker with a syringe every 0.5 h in order to maintain the concentration of CO_2_, and the ^13^C labeling lasted for 4 h. In order to keep low temperature during the labeling process, an appropriate amount of ice was added to the bottom of the labeling chamber to limit the temperature within the range of 28–37°C. At the same time, three other plants were selected as the control (^13^C natural abundance), and destructive samples were taken on the third day after labeling and ^13^C was determined.

Biomass samples for ^13^C and ^15^N analyses were collected at the end of the labeling for ^13^C and ^15^N analyses. The samples were dried and then ground with an electric grinder and filtered with a 0.25 mm mesh screen. The abundance of ^15^N and ^13^C were measured with a ZHT-03 mass spectrometer from the Beijing Analytical Instrument Factory (Chinese Academy of Agricultural Sciences). Three replicates were conducted for each treatment. The formula is calculated according to Wang et al. (2020).

Calculation of ^15^N

(1) Ndff(%)=abundance⁢of⁢N15⁢in⁢plant-natural⁢abundance⁢of⁢N15abundance⁢of⁢N15⁢in⁢fertilizer-natural⁢abundance⁢of⁢N15×100%

(2) N15⁢absorbed⁢by⁢each⁢organ⁢from⁢fertilizer⁢(mg)=Ndff(%)×Organtotalnitrogen(mg)

(3) N15distributionrate(%)=N15⁢absorbed⁢by⁢each⁢organ⁢from⁢fertilizer⁢(mg)total⁢N15⁢absorbed⁢by⁢plant⁢from⁢fertilizer⁢(mg)×100%

Ndff is the contribution rate of plant organ to total nitrogen from fertilizer of ^15^N

Calculation of ^13^C

(4) AbundanceofC13:F(%)i=(δ13⁢C+1000)×RPBD(δ13⁢C+1000)×RPBD+1000×100%R(standardratioofcarbonisotope)PBD=0.0112372

(5) $$\mathrm{Carbon}\mathrm{content}\mathrm{of}\mathrm{each}\mathrm{organ}:C{}_{i}=\mathrm{amount}\mathrm{of}\mathrm{dry}\mathrm{matter}\left(g\right)\times \mathrm{total}\mathrm{carbon}\mathrm{content}\left(\%\right)$$

(6) ContentofC13ofeachorgan:C13(mg)i=Ci×(Fi-Fnl)100×1000F:nlnoC13labeling,naturalabundanceofC13ofeachorgan

(7) C13distributionrate:C13(%)=Ci13Cnet⁢absorption13×100%

### Dry Matter Weight and K Content of Plant

After 30 days of treatment, the seedlings were divided into roots, stems, and leaves. The samples were washed in an order of tap water, detergent, deionized water, and 1% hydrochloric acid, and were then washed with deionized water for three times. The samples were dried at 105°C for 30 min, followed by 80°C for 3 days. The dry weight was recorded as the biomass. K content was measured according to Hu et al. (2017) using atomic absorption techniques. Three replicates were conducted for each treatment.

### Root Morphology and Root Activity

For each treatment group, three M9T337 seedlings were randomly sampled and rinsed with deionized water before analysis. The root images were analyzed with WinRhizo software (Regent Instruments Canada Inc). Total root length and total root surface area were calculated for each treatment.

Root activity was measured using the triphenyltetrazolium chloride (TTC) method described by Chen et al. (2018).

### Photosynthetic Parameters and Chlorophyll Fluorescence Parameters

After 30 days of treatment, the fourth main-stem leaf from the terminal of the plant were selected for measurement of photosynthetic parameters (*P*_n_, net photosynthetic rate; *G*_s_, stomatal conductance; *C*_i_, intercellular CO_2_ concentration). Every leaf was measured three times between 9:00 and 11:30 AM with a LI-6400XT portable photo synthesis system (LI-Cor, Lincoln, NE, United States).

During the same period, chlorophyll fluorescence parameters were estimated using a pulse modulated chlorophyll fluorescence meter (PAM 2500, Walz, Germany) on the same leaf. Every leaf was measured three times. The maximum photochemical quantum yield of PSII (*F*_v_/*F*_m_) was measured after a 25 min period of dark adaptation. Non-photochemical quenching (qN), coefficient of photochemical quenching (*q*P), and electron transport rate (ETR) were recorded after measuring light-adapted leaves.

### NO_3_^–^ Ion Flow Rate in Roots

The NO_3_*^–^* dynamic flow rate in roots was measured using a non-damaging micro-measurement system (NMT 100 Series, United States) at Shandong Agricultural University. The root system of M9T337 seedlings was washed with ultrapure water, and then the roots 1–2 cm away from the root tip were placed in a plastic dish with a filter paper strip, and the filter paper strip was fixed with a small glass block. The test solution [0.25 mM KNO_3_, 0.625 mM KH_2_PO_4_, 0.5 mM MgSO_4_, 0.25 mM Ca(NO_3_)_2_, pH 6.0] was added until the root was submerged. This was left to stabilize for 10 min, following which the test was started after the NO_3_^–^ flow rate on the root surface was stable. According to the measurement, the NO_3_^–^ ion flow velocity of seedlings from the different treatments was the largest in the dense root hair area about 8 mm from the root tip. Thus, each sample was randomly selected for a repeated test, and the data were collected at three points, where each treatment detected six samples, and each sample was stable for 10 min. After the test, MageFlux (imFluxes V2.0), a data analysis software provided by Xuyue company, was used for analysis. If the flow velocity J is positive, it indicates ion outflow, and if the flow velocity is negative, it indicates ion inflow.

### NR, GS, Rubisco, SPS and SS Activities

The NR (EC 1.6.6.1) and GS (EC 6.3.1.2) activities were measured according to the modified method described by Hu et al. (2016b). The supernatant was used for the measurement of light absorption at 540 nm, the NR activity was calculated from the nitrite N standard curve, and the GS activity was calculated from the standard curve of c-glutamyl hydroxamate.

The Rubisco (EC 4.1.1.39), SPS (EC 2.4.1.14), and SS (EC 2.4.1.13) activities were extracted according to Hu et al. (2016a). Three replicates were conducted for each treatment.

### RNA Extraction and Gene Expression Analysis

After 30 days of treatment, 0.1 g root was quickly ground into powder in liquid nitrogen. RNA was extracted by TRIzol reagent (Invitrogen). RNA integrity was detected by electrophoresis, and RNA purity was determined by a nucleic acid analyzer. The cDNA was reverse-transcribed using a 5 × all-in-one RT Master Mix (ABM, Canada) kit. According to the *NRT1.1* and *NRT2.1* gene sequence of the apple database,^1^ gene-specific primers were developed using Primer 3.0 (Table 1) based on the cDNA template. The *Actin* gene was used as a reference in accordance with the EvaGreen Express 2 × qPCR Master Mix (ABM, Canada) kit steps. the Real-time fluorescent quantitative PCR amplification was used to assess gene transcription. The reaction procedure was as follows: pre-denaturation at 95°C, 30 s; denaturation at 95°C for 5 s, annealing at 60–65°C for 30 s, extension at 72°C for 20–30 s, 45 cycles. All PCR reactions were performed with three biological and three technical replicates. The 2^–ΔΔ*CT*^ method was used for analyzing the real-time fluorescent quantitative PCR amplification data.

**TABLE 1:** Primer sequences for qRT-PCR.

Gene name	Forward sequence of the primers (5′→3′)	Reverse sequence of the primers (5′→3′)
*MdNRT1.1*	CTCGGCCTCATTGTGTTCTT	TCCAACGGCAGTTCCATATTC
*MdNRT2.1*	GCTGTACTCTTCCTGTGACTTT	CGTCGACTTCTCGACATCTTT
*Actin*	TGGTGTCATGGTTGGTATGG	CCGTGCTCAATGGGATACTT

### Statistical Analysis

Origin 8.0 (OriginLab Corporation, Northampton, MA, United States) was used to prepare the figures. Data were analyzed with SPSS 17.0 (SPSS, Inc., Chicago, IL, United States) using one-way factorial analysis of variance (ANOVA). In all cases, differences were considered significant at a probability level of *P* ≤ 0.05.

## Results

### Changes of Total Dry Matter, Root-Shoot Ratio and Root Activity

The biomass of each organ and the root-shoot ratio of M9T337 seedlings differed significantly under different K levels. As shown in Table 2, the biomass of seedlings was inhibited by low and high K treatments, and the highest was related to K6 treatment. With an increase in K supply, the root-shoot ratio initially increased and then decreased, and maximized under 6 mM K treatment, indicating that K deficiency and excessive K has a significant impact on root growth than that on aboveground parts of seedlings.

**TABLE 2:** Plant biomass and root/shoot ratio of M9T337 seedlings treated with K0 (0 mM K^+^), K3 (3 mM K^+^), K6 (6 mM K^+^), K9 (9 mM K^+^), or K12 (12 mM K^+^) in 2018 and 2019.

Treatment	2018			2019		
	Shoot dry weight (g/plant)	Root dry weight (g)	Root-shoot ratio	Shoot dry weight (g/plant)	Root dry weight (g)	Root-shoot ratio
K0	3.45 ± 0.25c	1.27 ± 0.08d	0.37 ± 0.01b	3.93 ± 0.14d	1.45 ± 0.08d	0.37 ± 0.03c
K3	4.03 ± 0.07bc	1.59 ± 0.08c	0.40 ± 0.02ab	4.30 ± 0.16c	1.77 ± 0.09c	0.41 ± 0.03ab
K6	5.07 ± 0.58a	2.17 ± 0.13a	0.43 ± 0.03a	5.37 ± 0.15a	2.30 ± 0.13a	0.43 ± 0.01a
K9	4.40 ± 0.21b	1.86 ± 0.12b	0.42 ± 0.01a	4.84 ± 0.15b	1.94 ± 0.07b	0.40 ± 0.00ab
K12	3.69 ± 0.98c	1.40 ± 0.08d	0.38 ± 0.02b	4.34 ± 0.27c	1.66 ± 0.10c	0.38 ± 0.01bc

*Each treatment had three biological replicates and values are expressed as means ± standard deviations (SD, *N* = 3). Different letters denote significant differences (*P* < 0.05).*

**FIGURE 1:**
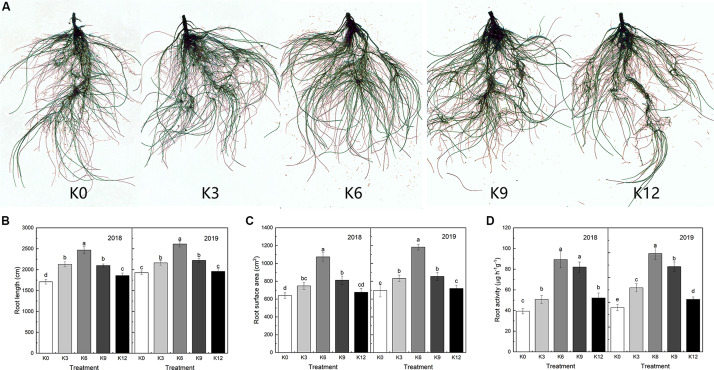
Root morphology **(A)**, root length **(B)**, root surface **(C)**, and root activity **(D)** of M9T337 seedlings treated with K0 (0 mM K^+^), K3 (3 mM K^+^), K6 (6 mM K^+^), K9 (9 mM K^+^), or K12 (12 mM K^+^) in 2018 and 2019. Each treatment had three biological replicates and the assays were repeated three times. Vertical bars indicate ± SD (*N* = 3). Different letters indicate statistically significant differences (*P* < 0.05).

The root morphology of M9T337 seedlings was also significantly affected by different K levels (Figure 1A). In 2018 and 2019, the root length and total root surface area of seedlings were the largest under 6 mM K concentration, and the root length and surface area of seedlings under 0 mM and 12 mM K treatment were significantly lower than those of the 6 mM treatment. The result showed that low and high K supply levels inhibited the elongation and growth of root (Figures 1B,C).

As shown in Figure 1D, K levels also significantly affected root activity. The highest root activity appeared in 6 mM K treatment, and inappropriate K levels had a negative effect on root activity.

### Changes of K Accumulation in Various Organs of M9T337 Seedlings

**FIGURE 2:**
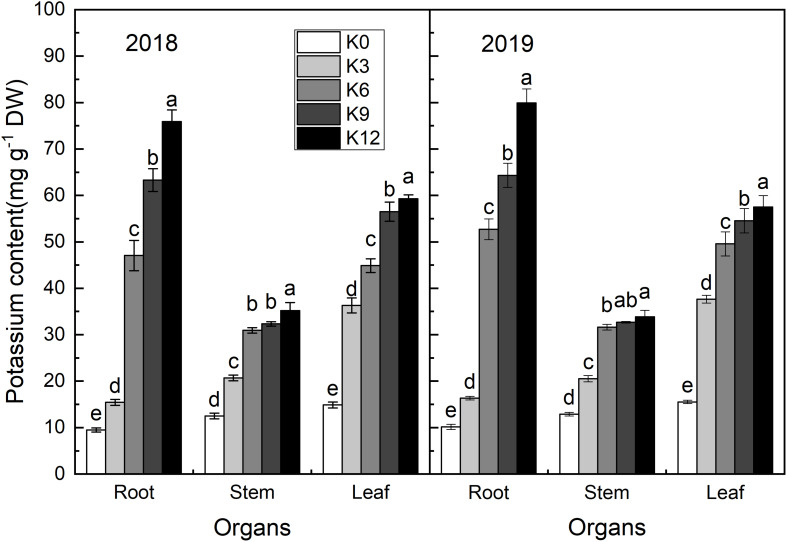
K concentrations (mg g^–1^ DW) in the leaves, stems, and roots of M9T337 seedlings treated with K0 (0 mM K^+^), K3 (3 mM K^+^), K6 (6 mM K^+^), K9 (9 mM K^+^), or K12 (12 mM K^+^) in 2018 and 2019. Each treatment had three biological replicates and the assays were repeated three times. Vertical bars indicate ± SD (*N* = 3). Different letters indicate statistically significant differences (*P* < 0.05).

Potassium content in the roots, stems, and leaves of seedlings increased with the increasing in K supply (Figure 2). The K content of all organs was the highest under K12 treatment. In K0 and K3 treatments, the K content of each organ was the highest in leaves, second-highest in the stems, and lowest in roots. Under the other K level treatments, the K content of each organ was highest in the roots and lowest in the stems. So, K was preferentially supplied to the shoot under low K conditions.

### Changes of Photosynthesis and Chlorophyll Fluorescence

We monitored photosynthetic rate and gas exchange parameters to determine possible effects of different K levels. With increasing K supply, *P*_n_ and *G*_s_ increased first and then decreased (Table 3), reaching the maximum value in K6 treatment. However, *C*_i_ was the highest in K0 treatment and the lowest in K6 treatment.

**TABLE 3:** Photosynthetic Parameters and chlorophyll fluorescence of M9T337 seedlings treated with K0 (0 mM K^+^), K3 (3 mM K^+^), K6 (6 mM K^+^), K9 (9 mM K^+^), or K12 (12 mM K^+^) in 2018 and 2019.

Year	Treatment	*P*_n_ [μmol(CO_2_) ⋅ m^–2^ ⋅ s^–1^)]	*G*_s_ [μmol(H_2_O) ⋅ m^–2^ ⋅ s^–1^]	*C*_i_ (μmol ⋅ mol^–1^)	*F*_v_/*F*_m_	ETR	*q*P	qN
2018	K0	9.6 ± 0.3d	121 ± 8d	262 ± 7a	0.773 ± 0.013c	145 ± 8d	0.733 ± 0.008d	0.953 ± 0.031a
	K3	11.3 ± 0.4bc	151 ± 7b	223 ± 7b	0.818 ± 0.015b	158 ± 4c	0.755 ± 0.012c	0.909 ± 0.014b
	K6	14.5 ± 0.5a	166 ± 9a	207 ± 10c	0.839 ± 0.009a	198 ± 11a	0.835 ± 0.018a	0.812 ± 0.010d
	K9	12.0 ± 0.4b	145 ± 5b	219 ± 8b	0.821 ± 0.013b	187 ± 8b	0.804 ± 0.012b	0.819 ± 0.021d
	K12	10.6 ± 0.4c	135 ± 5c	213 ± 9c	0.816 ± 0.011b	183 ± 12b	0.798 ± 0.010b	0.863 ± 0.008c
2019	K0	9.0 ± 1.4d	113 ± 6d	267 ± 10a	0.751 ± 0.014c	134 ± 10d	0.691 ± 0.022d	0.934 ± 0.022a
	K3	12.2 ± 0.4b	154 ± 9bc	237 ± 9b	0.815 ± 0.017b	179 ± 5c	0.783 ± 0.042b	0.887 ± 0.025b
	K6	16.3 ± 0.7a	188 ± 6a	203 ± 4d	0.841 ± 0.006a	211 ± 10a	0.857 ± 0.021a	0.784 ± 0.014d
	K9	12.4 ± 0.6b	164 ± 9b	225 ± 3c	0.825 ± 0.008b	193 ± 11b	0.797 ± 0.031b	0.807 ± 0.021c
	K12	10.4 ± 0.3c	148 ± 7c	204 ± 7d	0.809 ± 0.011b	178 ± 7c	0.742 ± 0.018c	0.865 ± 0.008b

*Each treatment had three biological replicates and values are expressed as means ± SD (*N* = 3). Different letters denote significant differences (*P* < 0.05).*

Chlorophyll fluorescence was further investigated to understand the internal causes of the effects of different K levels on photosynthesis. The K6 seedlings had a significantly higher *F*_v_/*F*_m_, ETR and *q*P, and the qN was the lowest (Table 3). This indicates that unsuitable K levels can inhibit photosynthetic electron transfer, increase heat dissipation and even damage the light system (K0 treatment).

### Changes of Rubisco, SPS and SS Activities

**FIGURE 3:**
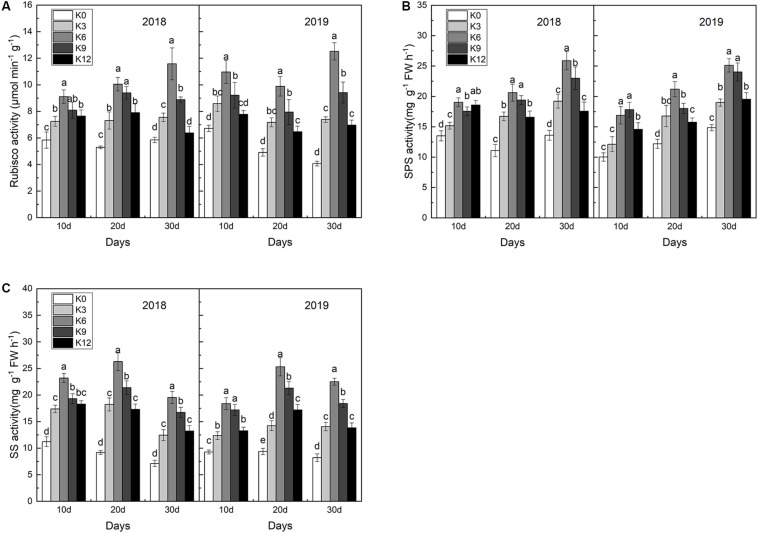
Rubisco **(A)**, SPS **(B)**, and SS **(C)** activities in the leaves of M9T337 seedlings treated with K0 (0 mM K^+^), K3 (3 mM K^+^), K6 (6 mM K^+^), K9 (9 mM K^+^), or K12 (12 mM K^+^) in 2018 and 2019. Each treatment had three biological replicates and the assays were repeated three times. Vertical bars indicate ± SD (*N* = 3). Different letters indicate statistically significant differences (*P* < 0.05).

In order to explore the effect of K supply on C metabolizing enzymes, we measured the activities of Rubisco, SPS and SS in leaves. As shown in Figure 3, after 30 days, seedlings subjected to K6 treatments acquired the highest activities of Rubisco, SPS and SS, followed by K9 treatments, and K0 treatments obtained the lowest activities. These results indicate that low or high K levels can significantly inhibit the activity of C metabolizing enzymes in leaves, and the inhibition effect is more significant under low K levels than that under high K levels.

### Changes of ^13^C Accumulation and Distribution

**FIGURE 4:**
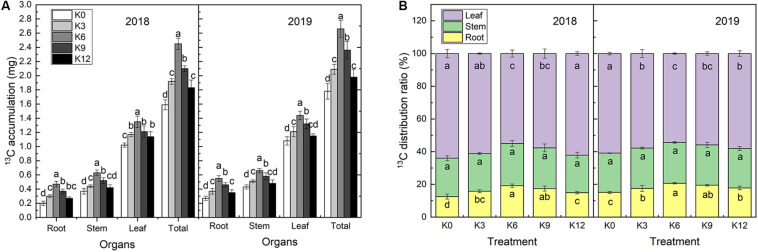
^13^C accumulation **(A)** and ^13^C distribution ratio **(B)** of M9T337 seedlings treated with K0 (0 mM K^+^), K3 (3 mM K^+^), K6 (6 mM K^+^), K9 (9 mM K^+^), or K12 (12 mM K^+^) in 2018 and 2019. Each treatment had three biological replicates and the assays were repeated three times. Vertical bars indicate ± SD (*N* = 3). Different letters indicate statistically significant differences (*P* < 0.05).

^13^C isotope labeling results showed that ^13^C accumulation in M9T337 seedlings significantly varied under different levels of K supply (Figure 4). The ^13^C accumulation rate in all organs of seedlings under the K6 treatment were significantly higher than those under of other treatments (Figure 4A).

The distribution ratio of ^13^C to each organ is related to its competitive ability but also chiefly to its transport capacity within the plant. The ^13^C distribution rates for each treatment were consistent in both years, among which the leaves had the highest values followed by the stems, and roots (Figure 4B). K supply increased the ^13^C distribution rate in roots, which increased first and then decreased with increasing K levels. The ^13^C distribution rate in roots treated with 6 mM K^+^ was the highest and increased by 52.65% and 36.58% compared with the K0 treatment in 2018 and 2019, respectively. An different trend was, however, observed for leaves. No significant effect was observed on the ^13^C distribution ratio in stems under K treatments.

### Changes of NO_3_^–^ Ion Flow Rate in Roots

**FIGURE 5:**
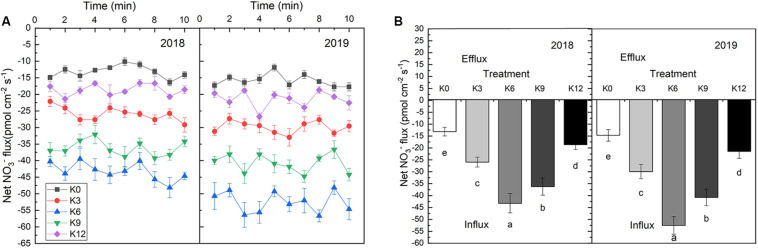
NO_3_^–^ ion flow rate in root of M9T337 seedlings treated with K0 (0 mM K^+^), K3 (3 mM K^+^), K6 (6 mM K^+^), K9 (9 mM K^+^), or K12 (12 mM K^+^) in 2018 and 2019. **(A)** Net NO_3_^–^ fluxes in the root of apple seedlings for 10 min. **(B)** Mean rate of NO_3_^–^ fluxes during the entire 10 min. Data are means ± SD (*N* = 6). min = minutes. Different letters indicate statistically significant differences (*P* < 0.05).

The NO_3_^–^ ion flow velocity in roots increased at first as K supply rose, but then decreased, which implied that a moderate K supply level will promote nitrate ions to be take up by roots (Figure 5). In 2018 and 2019, the average NO_3_^–^ flux rates within 10 min of seedling root exposure to K6 treatment increased by 229.3% and 132.1%, 256.7%, and 144.7%, respectively, compared with those under K0 and K12 treatments.

### Changes of Nitrogen-Metabolizing Enzymes Activities

**FIGURE 6:**
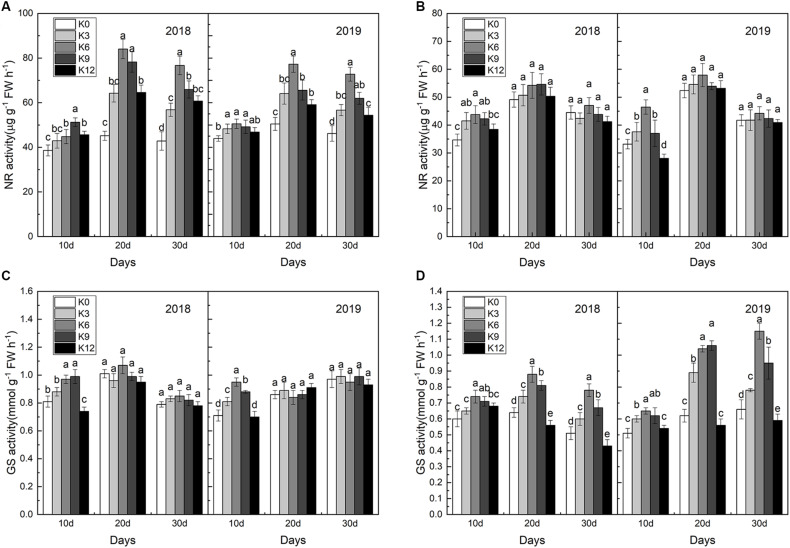
NR activities in the leaves **(A)** and roots **(B)**; GS activities in the leaves **(C)**, and roots **(D)** of M9T337 seedlings treated with K0 (0 mM K^+^), K3 (3 mM K^+^), K6 (6 mM K^+^), K9 (9 mM K^+^), or K12 (12 mM K^+^) in 2018 and 2019. Each treatment had three biological replicates and the assays were repeated three times. Vertical bars indicate ± SD (*N* = 3). Different letters indicate statistically significant differences (*P* < 0.05).

Compared with roots, NR activities in leaves were significantly affected by K levels (Figure 6), and NR activities in leaves under the K6 treatment were significantly higher than the other treatments for all three stages (Figure 6A), which improved nitrate assimilation capacity. In contrast, NR activities in the leaves under the K0 and K12 treatments were relatively lower. Unlike NR activities, differences in GS activities in roots was greater between different K treatments than that in leaves (Figures 6C,D), and the higher values appeared in the K6 treatment.

### Changes of Expression of NRT1.1 and NRT2.1 in Roots

**FIGURE 7:**
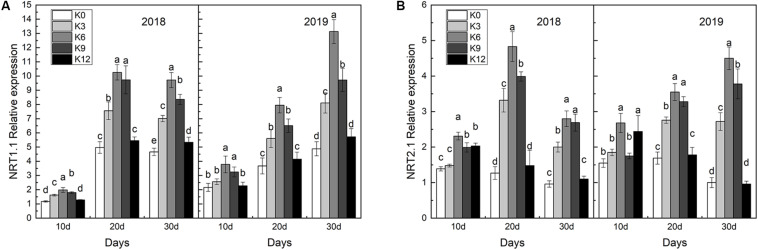
*NRT1.1* gene expressions **(A)** and *NRT2.1* gene expressions **(B)** in the roots of M9T337 seedlings treated with K0 (0 mM K^+^), K3 (3 mM K^+^), K6 (6 mM K^+^), K9 (9 mM K^+^), or K12 (12 mM K^+^) in 2018 and 2019. Each treatment had three biological replicates and the assays were repeated three times. Vertical bars indicate ± SD (*N* = 3). Different letters indicate statistically significant differences (*P* < 0.05).

We compared the effects of the different K supply levels on *NRT1.1* and *NRT2.1* gene expressions and discovered that their expressions were affected by K applications (Figure 7). As expected, we found that *NRT1.1* and *NRT2.1* gene expressions in roots were significantly higher under the K6 treatment compared to the control (K0) and the excessive K supply treatment (K12), which suggested that K can induce the nitrate-responsive pathway for NRT gene family expression in M9T337 seedlings.

### Changes of ^15^N Absorption, Distribution, and Use Efficiency

**FIGURE 8:**
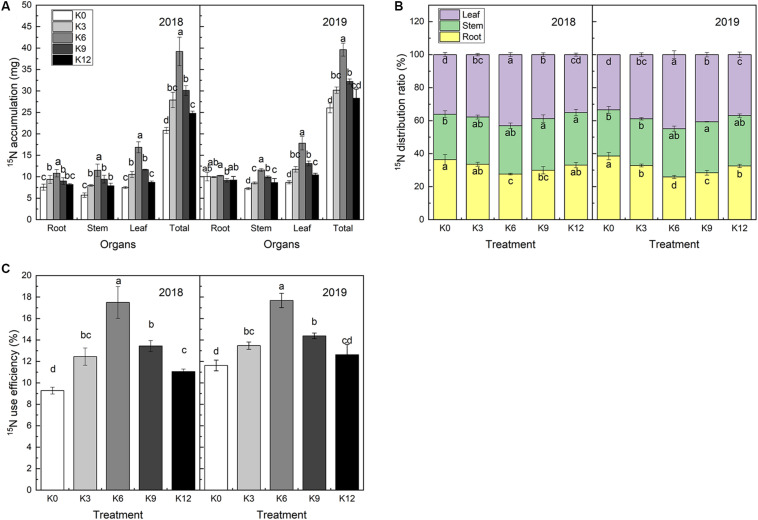
^15^N accumulation **(A)**, ^15^N distribution ratio **(B)** and ^15^NUE **(C)** of M9T337 seedlings treated with K0 (0 mM K^+^), K3 (3 mM K^+^), K6 (6 mM K^+^), K9 (9 mM K^+^), or K12 (12 mM K^+^) in 2018 and 2019. Each treatment had three biological replicates and the assays were repeated three times. Vertical bars indicate ± SD (*N* = 3). Different letters indicate statistically significant differences (*P* < 0.05).

^15^N isotope labeling results showed that there were significant differences in ^15^N absorption and ^15^N use efficiency of M9T337 seedlings under different K levels (Figures 8A,C). After 30 days of treatment, the ^15^N absorption rate of seedlings in K6 treatment was significantly higher than that of the other treatments, and the 2-year average was 1.70 and 1.49 times higher than that of K0 and K12 treatments, respectively.

The ^15^N use efficiency was calculated from the ^15^N absorption content divided by the total ^15^N application rate. As expected, ^15^N use efficiency was highest in K6 treatment and lowest in K0 treatment (Figure 8C). This declared that the allocation of NO_3_^–^ to the roots/leaves might be responsible for the difference in ^15^N use efficiency among the different K supply levels treatments.

## Discussion

### Effects of K Levels on Root Growth and ^13^C Assimilation and Distribution of M9T337 Seedlings

Potassium has a significant effect on the growth and development of plant roots. Jung et al. (2009) found that in *Arabidopsis thaliana*, root hair elongation could be promoted in a K-deficient environment after 28 h. Høgh-Jensen and Pedersen (2003) and Jilani (2008) discovered that pea (*Pisum sativum*), barley (*Hordeum vulgare*), perennial ryegrass (*Lolium perenne*), rye (*Secale cereale*), and red clover (*Trifolium pratense*) could modify their root hair length in response to low K conditions. Therefore, plants can cope with short-term K deficiency by promoting root growth. However, compared with the lack of N and phosphorus, the growth of plant roots is strongly inhibited under conditions of prolonged K deficiency, and the root-shoot ratio will be significantly reduced (Hermans et al., 2006; Chen et al., 2015). We also found that moderate K concentrations (6 mM K^+^) can promote root growth. However, root growth was significantly inhibited by unsuitable K supply levels, which may be due to an increase in ethylene and a decrease in indole-3-acetic acid (IAA) in the roots (Zhang et al., 2009). At the same time, the limited transport of photosynthetic products from leaves to roots may also explain why unreasonable K supply hinders root growth (Figure 4B).

The percentage of ^15^N in each organ accounting for the total ^15^N content reflects the distribution of N fertilizer in the seedlings and the migration regularity in the organs. ^15^N distribution ratio in roots was found higher in K0 and K12 treatments, while the lowest in K6 treatment. And, the opposite trend exhibited in the leaves (Figure 8B). This suggested that the K level can affect root-to-leaf transportation.

Potassium status of plants has a significant effect on the transport and distribution of photosynthetic products (Pettigrew, 2008). Sufficient K supply can establish osmotic potential in the phloem and help to transfer photosynthates from source to sink organs (Cakmak, 2005). However, the loading of photosynthates in phloem of K deficient plants is inhibited and the transport to roots is significantly reduced (Gerardeaux et al., 2010). In this study, the ^13^C isotope labeling results showed that the ^13^C assimilation rate and distribution ratio in roots were higher under an appropriate K supply level. This indicates that insufficient or excessive K supply can inhibit the C assimilation of leaves and the photosynthetic products transport from leaves to roots. Correlation analysis also showed that the ^13^C distribution ratio was positively correlated with root biomass. Meanwhile, the ^13^C distribution ratio of roots was significantly positive related to photosynthesis and C metabolizing enzyme activity (SS and SPS activities). We also measured the gas exchange parameters of leaves, and found that the *P*_n_ and *G*_s_ of seedling decreased significantly under the treatment of low and high K supply, which indicates that inappropriate K supply would limit photosynthesis through stomatal restrictions. *C*_i_ increased significantly under K0 treatment, therefore, the decrease of *P*_n_ may also be related to limitations of the optical system. Furthermore, we found that *F*_v_/*F*_m_, *q*P and ETR of leaves under insufficient or excessive K supply were significantly lower than those of the 6 mM K treatments. These results clearly show that K deficiency or excess can inhibit the photochemical efficiency and electron transfer efficiency of PSII reaction center. Lu et al. (2016) also found similar results on oilseed rape. In conclusion, we showed that K affected C assimilation and distribution by regulating photosynthesis and C metabolizing enzymes. The increase in the distribution of photosynthetic products to the root system will promote growth and development of the root system, then improving the absorption ability of N, thus increasing NUE.

### Effect of K Levels on Nitrate Metabolism of M9T337 Seedlings

Previous research has indicated that a close relationship exists between K^+^ and root-induced NO_3_^–^ uptake (Dong et al., 2004). Parker and Newstead (2014) showed that *NRT1.1* can take up nitrate using two distinct affinity modes. Li et al. (2007) and Munos et al. (2004) demonstrated that *NRT2.1* is a high-affinity transporter for nitrate. We tested the responses of M9T337 seedlings to different concentrations of K and found that 6 mM K^+^ induced the greatest increase in *NRT1.1* and *NRT2.1* expressions, which led to a higher nitrate absorption capacity. Meanwhile, a higher net NO_3_^–^ influx rate under 6 mM K^+^ treatment also implied that a moderate K supply level will promote nitrate ions into roots (Figure 5). In addition, an ideal root morphology and activity were important for nutrient absorption (Sattelmacher et al., 1994). Our results also found that M9T337 seedlings treated with 6 mM K^+^ had larger root surface area and higher root activity. As a result, a higher ^15^N absorption content appeared under appropriate K supply conditions (Figure 8). In addition, K also affected the distribution of NO_3_^–^ between root and shoot (Ruiz and Romero, 2002). Our results show that higher ^15^N distribution ratio in roots were found in K deficient or excess treatments, while the highest ^15^N distribution ratio in leaves appeared under appropriate K supply treatments. This suggests that appropriate K supply not only increases NO_3_^–^ absorption in roots, but also promotes the transport from roots to shoots. Rufty et al. (1981) and Wang et al. (2003) also reported that K deficiency can seriously hinder the assimilation and translocation of nitrate, and higher N assimilation occurs in the roots. So it is necessary to focus on the effect of K on N assimilation in roots and leaves.

Nitrate reductase is the key enzyme for regulating the rate limiting step of the NO_3_^–^ assimilation pathway (Kovács et al., 2015; Ren et al., 2017; Teng et al., 2017). NO_3_^–^ will be transformed into NH_4_^+^ in plants, and NH_4_^+^ will be converted mainly through the GS/GOGAT pathway. Thus the activity of NR and GS will affect the absorption and assimilation of N. The inhibition of K deficiency on NR activity has been verified in cotton, cucumber, and *Arabidopsis* (Ruiz and Romero, 2002; Balkos et al., 2010; Hu et al., 2016b). Generally, with high external K^+^ supply, the co-translocation of K^+^ and NO_3_^–^ to the shoot increases (Ben Zioni et al., 1971; Blevins et al., 1978), and both storage of NO_3_^–^ and NR activity increases in leaves, while less N assimilation is found in roots (Blevins et al., 1978; Rufty et al., 1981). Consistent with previous results, we also found that for a certain range, with the increase of K supply, GS activity of roots and NR activity of leaves of M9T337 seedlings gradually increased, which promoted the assimilation of NO_3_^–^. However, when the K supply is too high, the activity of these enzymes will decrease, which may be related to the inhibition of photosynthesis and the reduction of energy supply.

The absorption and distribution of nitrate also depends on the energy and C skeleton from photosynthesis (Liu et al., 2017; Yin et al., 2019). We found that the higher NUE was related to the improvement of the fixation rate of photosynthetic carbon and the efficiency of photosynthetic electron transfer under the appropriate K supply level. In addition, the results of ^15^N labeling showed that low or high K levels were not conducive to NO_3_^–^ transport from root to leaf, which was consistent with previous studies (Ruiz and Romero, 2002; Coskun et al., 2016). Han et al. (2016) found that the increased distribution of NO_3_^–^ to the aboveground parts will enable crops to fully utilize sunlight energy to carry out NO_3_^–^ metabolism and energy conversion, so as to improve the NUE of crops. Perchlik and Tegeder (2018) also reported that increasing N allocation to leaves represents an effective strategy for improving C fixation and photosynthetic nitrogen use efficiency (NUE).

Consequently, the M9T337 seedlings treated with 6 mM K^+^ showed better growth, improved photosynthate production and NUE. Our findings reveal how the application of K affects the uptake, transport, and assimilation of NO_3_^–^, and deepens our understanding of the relationship between K supply and improved NUE. It should be noted that our research was mainly focused on physiological mechanisms on K induces positive changes in N metabolism in M9T337 seedlings. Further molecular studies are needed to gain a deeper understanding of how K improves NUE.

## Conclusion

Our results showed that M9T337 seedlings treated with the optimum K levels had (i) enhanced ^13^C accumulation and ^13^C transport from leaves to roots; (ii) increased root NO_3_^–^ ion flow rate; (iii) relatively high N metabolic enzyme activities; (iv) up-regulated transcript levels of nitrate uptake genes (*NRT1.1*; *NRT2.1*); (v) enhanced ^15^N translocation from roots to leaves; (vi) higher ^15^NUE. In conclusion, optimum K levels can increase NUE by affecting root morphology and activity, the activity of enzymes involved in C and N metabolism, nitrate uptake genes, and nitrate transport.

## Data Availability Statement

All datasets generated for this study are included in the article/supplementary material.

## Author Contributions

YJ, SG, and XX conceived and designed the experiments. XX, XD, FW, JS, QC, GT, and ZZ performed all the experiments. XX, ZZ, and SG analyzed the data and wrote the manuscript. All authors contributed to the article and approved the submitted version.

## Conflict of Interest

The authors declare that the research was conducted in the absence of any commercial or financial relationships that could be construed as a potential conflict of interest.

## Footnotes

1 https://www.rosaceae.org/
